# Navigated Recovery of Fractured Dental Injection Needles: Case Report and Suggestions for Management during Pandemic Crises

**DOI:** 10.1155/2021/8820381

**Published:** 2021-01-16

**Authors:** Lara Schorn, Christoph Sproll, Rita Depprich, Norbert R. Kübler, Majeed Rana, Daman D. Singh, Julian Lommen

**Affiliations:** Department of Oral, Maxillofacial and Facial Plastic Surgery, University Hospital Düsseldorf, Düsseldorf, Germany

## Abstract

Dislocation of a fractured hypodermic needle is a complication requiring immediate and adequate emergency treatment. In this case report, 3D navigation is evaluated for its use to recover a quickly moving fractured needle. The needle was recovered safely, but it could be demonstrated that navigational planning has to be conducted right before surgery and other navigational tools, such as ultrasound, should be considered as well. Furthermore, an approach is suggested for treatment during pandemic crises such as COVID-19.

## 1. Introduction

In dental surgery, local anesthesia is tremendously important for a painless therapy. It is usually associated with low overall risks and complications [1]. Systemic reactions are for example hypertension, circulatory collapse, and toxic or allergic reactions [2]. Local events like hemorrhage, wound infections, soft tissue damage, nerve irritation, and dislodgement of fractured hypodermic needles can be observed [3]. There are various reasons for needle breakage. Most commonly, fatigue fractures of the needles occur [2]. These incidents, however, have been reduced after the introduction of disposable cannulas in the 1970s. Fractured needles can lead to serious and potentially life-threatening consequences by damaging adjacent vital anatomical structures [4, 5]. Quick extraction of the broken fragment is generally recommended (with very few exceptions). Recovery rates have been reported to be around 95% [2] with little postoperative complications. A recent review of Acham et al. [2] imposed a workflow including 3D navigation as an option for needle removal.

The outbreak of the global pandemic of coronavirus-disease 2019 (COVID-19) is changing medical and surgical procedures. An important task is to reduce the likelihood of infection for patients and medical staff [6]. Dentists, maxillofacial surgeons, and ENT surgeons are particularly exposed due to high viral load in the nasal cavity of infected patients [7]. Since a dislodged dental needle can easily become a life-threatening emergency, an adequate procedure for treatment under pandemic conditions is necessary.

In this case report, 3D navigation is used in a university hospital setting to recover a dislodged fractured needle, and a procedure for pandemic emergencies is suggested.

## 2. Case Presentation

**Figure 1:**
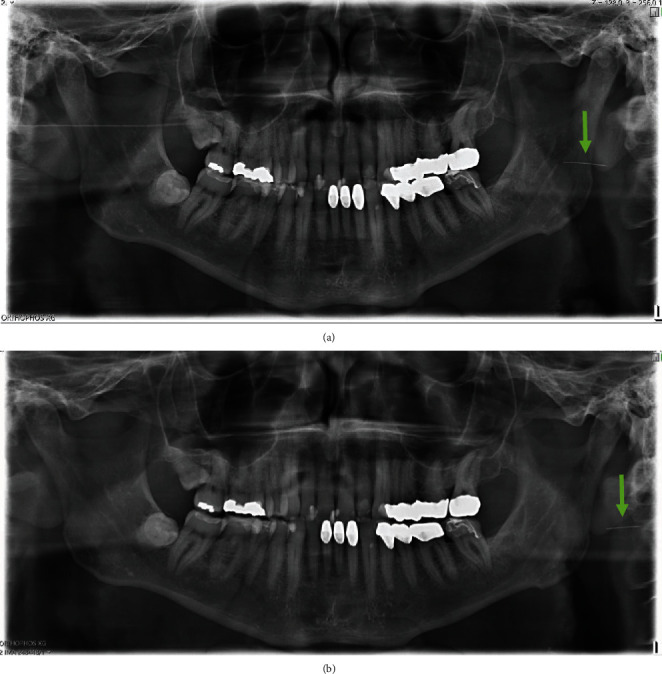
Orthopantomographic images on the day of initial presentation (a) and one day afterwards (b). The dislodged fractured hypodermic needle is located at the left ascending mandibular ramus and moved by about 1.5 cm after one day.

**Figure 2:**
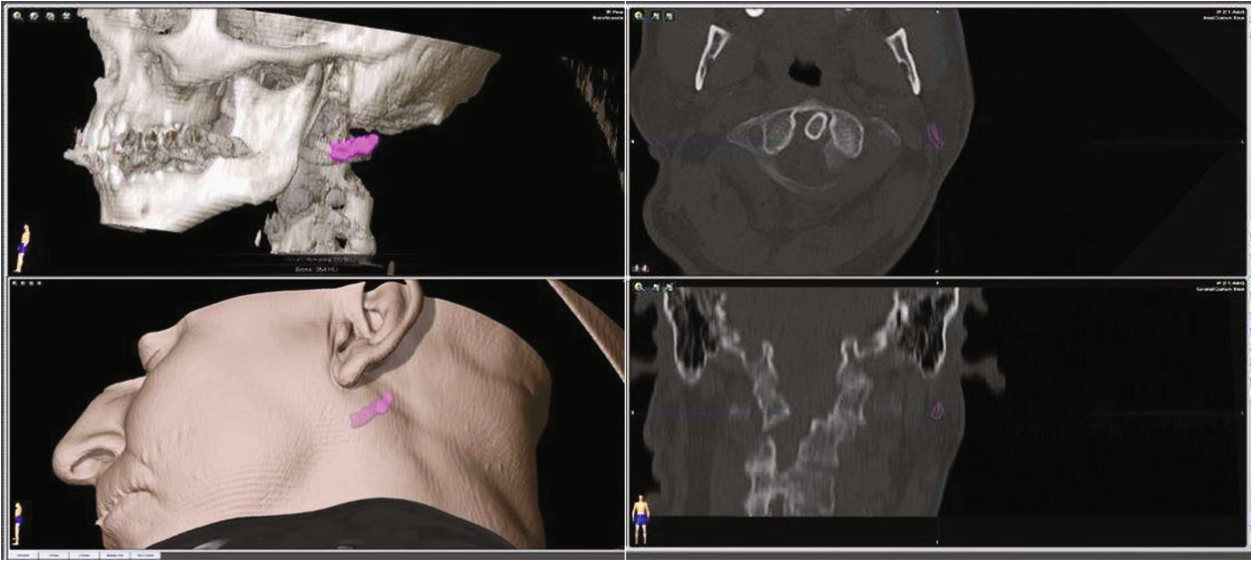
3D-location of the fractured hypodermic needle.

A 61-year-old, otherwise healthy, male Caucasian patient was referred by his local dentist to our clinic with a 30 G broken needle after anesthesia with inferior alveolar nerve block technique at his local dentist. The patient was stable and did not experience any pain or discomfort. An immediately taken orthopantomogram (OPTG) together with a cephalometric image and cone beam computer tomography (CBCT) showed the needle at the medial side of the ascending ramus close to the mandibular foramen (Figure 1). The patient was planned for removal by an intraoral approach in general anesthesia for the next day. Laboratory testing was done showing no pathological results. No medication was administered. On the morning of the next day, another OPTG was taken to confirm needle position. It showed lateral movement of the needle by around 1.5 cm (Figure 1). Therefore, a CT (SOMATOM Flash CT) with 0.75 mm slice thickness was taken and intraoperative 3D navigation was planned using iPlan 3.0.5 (BrainLab®, Feldkirchen, Germany) (Figure 2). The CT showed needle position to be retroauricular approximately 2 cm dorsal and 1.5 cm below the left ear lobe. Immediately afterwards, the patient was prepped for surgery and told not to move his head. General anesthesia was performed using remifentil (2 mg/Nacl 30 ml/h; Ultiva®, GlaxoSmithKline, Brentford, United Kingdom), propofol 1% 10 mg/ml 160 ml (B. Braun Melsungen AG, Melsungen, Germany), and recuromium bromide 10 mg/ml 30 ml (B. Braun Melsungen AG, Melsungen, Germany). Oral intubation was performed without any complications.

### 2.1. Surgical Procedure

**Figure 3:**
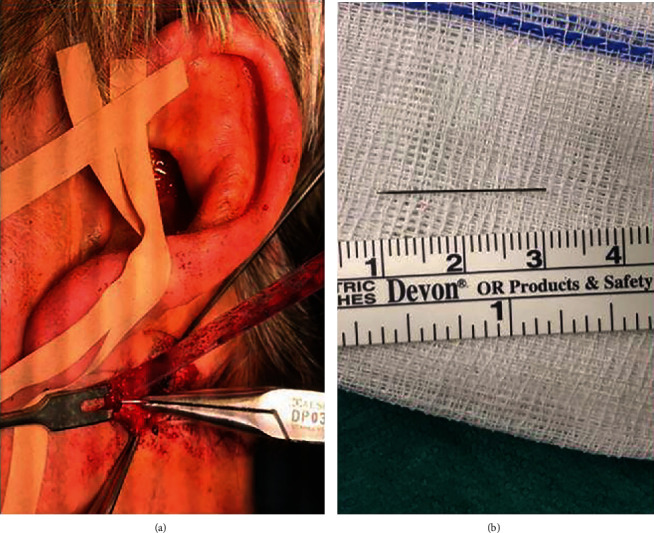
Intraoperative retrieval of the 2.1 cm fractured hypodermic needle (a) and measurement afterwards (b).

At first, an incision on the left parietal side of approximately 1.5 cm down to the periosteum was made. Denudation of the calvaria over an area of approximately 1 cm^2^ and predrilling with a 5 mm long 1.1 twist drill followed. Then, the scull reference array (tripod) was inserted into the patient's scull and fixed with an 8 mm screw (Medartis AG, Basel, Switzerland). The earlier constructed dental splint with four attached registration screws was inserted. After registration verification, the needle could be located by 3D navigation (Kolibiri Navigation System Cranial 3.0, Brainlab®, Feldkirchen, Germany) with an accuracy of 0.7 mm. The surgical approach was minimally invasive by extraoral incision below the left earlobe. At first, the skin and subcutis, as well as the musculature, were cut using a No. 10-blade scalpel. Hemostasis was performed by electrocoagulation. Then, the parotid capsule was dissected, and the underlying tissue was very carefully dissected layer by layer until the cannula tip could be identified. The 2.1 cm long needle could be fully recovered (Figure 3). Afterwards, the parotid capsule and the musculature injured by the cannula was reconstructed (reconstruction of the sternocleidomastoid muscle and the platysma with Vicryl4 × 0(Ethicon, Johnson & Johnson, Somerville, USA), a multilayer wound closure with Vicryl4 × 0, and finally a simple skin flap plastic with Ethilon5 × 0(Ethicon, Johnson & Johnson, Somerville, USA) were performed). Then, removal of the tripod, reconstruction of the occipitofrontal muscle with Vicryl 3 × 0 (Ethicon, Johnson & Johnson, Somerville, USA), and skin suture with Prolene 3 × 0 (Ethicon, Johnson & Johnson, Somerville, USA) followed. Operation time was 32 minutes.

Postoperatively, the patient showed no signs of complications, in particular no hemorrhage or infection. In a follow-up visit 10 days after the operation, no signs of infection, wound dehiscence, or any other recognizable pathologies could be seen. The sutures were removed, and further follow-up visits were performed by his local dentist. There were no adverse and unanticipated events. Written informed consent was obtained to publish his case.

### 2.2. Treatment during Pandemics

**Figure 4:**
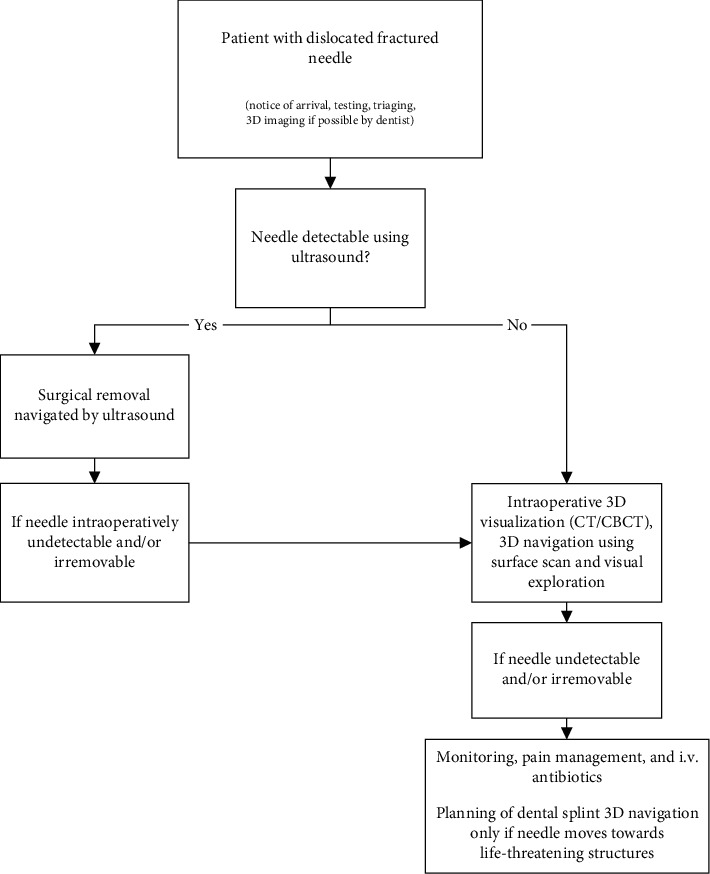
Suggestion for treatment during pandemic crises such as COVID-19. Dislocated fractured needle movement usually requires treatment in a department for maxillofacial surgery.

The case described above happened only one month before the outbreak of COVID-19. During the outbreak, we realized that when fractured needles are recovered as described, the risk of infection for patients and staff is high. We therefore also present a procedure reducing infective transmission to a minimum (Figure 4). The dentist transferring the patient should contact the maxillofacial department before sending the patient and ideally provide 3D imaging. Upon arrival, the patient should be separated, triaged, and tested. Afterwards, needle detection by ultrasound should be tried, using adequate COVID-19 protection gear, such as respirators of a high protection level (FFP3/N99/equivalent), waterproof gowns, eye protection, and gloves [7]. If testing is possible, test results should be awaited. If the needle can be located by ultrasound, sonography-controlled surgical needle recovery should be performed. If the needle cannot be located using sonography, intraoperative 3D visualization such as CT or CBCT should be used in combination with visual exploration, preferably using an extraoral approach. In cases with difficulties finding the needle, 3D navigation by surface scan could follow. If the needle still remains undetectable and/or irremovable, the patient should be closely monitored. Complex highly accurate 3D navigation with dental splints should only be planned if the needle moves towards a potentially life-threatening structure, such as the carotid artery, under strict infection-controlled measures.

## 3. Discussion

The presented case demonstrates a fractured dental syringe needle advancing from the pterygomandibular space towards the retroauricular region. We applied the treatment algorithm recently suggested by Acham et al. [2] and retrospectively evaluated it for its feasibility during pandemics such as COVID-19. At first, we planned to remove the needle conventionally by intraoral incision, searching for it in its approximate location. After realizing the movement of the needle, we researched the literature for further options. Acham et al. [2] suggest in a recent review an algorithm for needle recovery using 3D navigation. We therefore chose to use computer-assisted surgery to extract the needle. With navigational support, it was very easy to find the needle that had only slightly moved after planning. It could be recovered by a minimally invasive extraoral retromandibular approach. However, the process to enable 3D navigation takes time for the dental splint to be constructed, for additional CT imaging, and for the foreign object to be marked in the software. This takes some time in which the needle can dislocate further. If we had virtually located the needle and planned the navigation after initial imaging, the navigation would have been useless during surgery. It might therefore be helpful to plan navigated extractions only directly before the surgery with the most recent imaging. In only around 50% of reported cases, the needle is removed within one day after the event [2]. In our case, a longer waiting period might have dislocated the needle even further. Brooks and Murphy reported a case in which a dislocated fractured needle had been retrieved from the right internal carotid artery [8]. Luckily, the needle in our case did not damage any vital structures and could be removed with a minimally invasive procedure. In addition, the delay of treatment by one day and preparation for navigation led to hospitalization of the patient in which he had close contact to medical personnel in the emergency unit, the maxillofacial ward, the radiology department, and dental technicians. 3D navigation is not available in every hospital yet, and hardware and software are expensive to obtain. Planning costs and efforts are immense. Additionally, irradiation exposure is high, because when not planned immediately, additional 3D imaging (usually CT) with a dental splint for registration is necessary. Alternatives for 3D navigation suggested in the workflow by Acham et al., when immediate recovery is not possible, are stereotactic localization and visual exploration. Stereotactic options, such as 3D marking by indicator needles, the use of a C-arm, and intraoperative 3D visualization (CT, CBCT) are associated with high irradiation as well. All these expenses may seem like overtreatment in some cases. Visual exploration alone, however, is often confusing and might lead to severe damage. Sonography might be an alternative worth discussing. As it was in our case, the majority of needle fractures occur with inferior alveolar and mandibular nerve block anesthesia, with fragments predominantly situated in the pterygomandibular region [2]. We did not consider using ultrasound for needle location because of sonographic shadowing of the mandibular bone. Besides, applied pressure for sonography might have dislocated the needle further. However, it might be an easy, cheap, and quick intraoperative solution for needle location and should be considered as an additional intraoperative tool especially in cases of fast dislocation. Needles can usually be detected easily within the soft tissue. In difficult cases, intraoperatively navigated 3D-ultrasound, as it is used in neurosurgery, might be advantageous [9]. In times when extra infection precaution procedures have to be applied, procedures should consume as little time and resources as possible and be easy to handle even for less experienced personnel. The suggested treatment plan offers a way around difficult and long planning and can be managed by only one maxillofacial surgeon, limiting the risk of infection. However, it has not yet been tested and requires good sonographic skills. It will be tested as soon as the opportunity presents itself. Other approaches might prove easier and more applicable to daily clinical routines.

The recovery of broken dental needles proves to be very elaborate. Prevention of needle breakage therefore is even more important.  Table 1 offers crucial points for avoiding needle breakage. In case a needle breaks and the fragment is still visible, it should immediately be grasped with, e.g., a forceps or a hemostat. The patient should remain as immobile as possible since the visible fragment may disappear with released tissue tension [10].

In conclusion, 3D navigation works well for recovering dislodged fractured hypodermic needles. However, fractured needles can move quickly. Therefore, early recovery is necessary and 3D navigational planning has to be conducted immediately before surgery. Nevertheless, costs, efforts, and risks for infection are high. Other options, such as ultrasound, should be evaluated pre- and intraoperatively, especially during pandemics such as COVID-19.

### 3.1. Patient's Perspective

The patient denied to offer his full perspective on the case but claims to have felt treated well during the procedure.

## Figures and Tables

**Table 1:** Eight crucial points for avoiding needle breakage [2].

(1)	Explore the patient-specific anatomy
(2)	Use a sufficiently strong and long canula
(3)	Inspect for damage before use
(4)	Avoid prebending or kinking
(5)	Prepare the patient for puncture and avoid sudden movements
(6)	Do not insert the whole needle
(7)	Puncture only when masticatory musculature is relaxed
(8)	Avoid directional change during needle advancement
(9)	Change the needle for every injection

## Data Availability

Data will be made available by the corresponding author upon reasonable request.
